# Comparison of Effectiveness of Programmed Death Protein 1 and Programmed Death Ligand 1 Inhibitors in Extensive-Stage Small-Cell Lung Cancer: A Meta-Analysis of Randomized Controlled Trials and Observational Studies

**DOI:** 10.7759/cureus.55654

**Published:** 2024-03-06

**Authors:** Nanush Damarpally, Divine Besong Arrey Agbor, Tanya Sinha, Kalyan Naik Gugulothu, Ye Kyaw Myint, Sandipkumar S Chaudhari, Danish Allahwala

**Affiliations:** 1 Health Sciences, Houston Community College, Houston, USA; 2 Internal Medicine, Richmond University Medical Center, Staten Island, USA; 3 Medical Education, Tribhuvan University, Kirtipur, NPL; 4 Internal Medicine, Sri Venkateswara Medical College, Tirupathi, IND; 5 Internal Medicine, University of Medicine 1, Yangon, MMR; 6 Cardiothoracic Surgery, University of Alabama at Birmingham, Birmingham, USA; 7 Family Medicine, University of North Dakota School of Medicine and Health Sciences, Fargo, USA; 8 Nephrology, Fatima Memorial Hospital, Karachi, PAK

**Keywords:** systematic review and meta-analysis, extensive-stage small-cell lung cancer, lung cancer, pd-l1 inhibitors, pd-1 inhibitors

## Abstract

This meta-analysis aimed to compare the efficacy of programmed death protein 1 (PD-1) inhibitors and programmed death ligand 1 (PD-L1) inhibitors in patients with extensive-stage small-cell lung cancer. The present meta-analysis was conducted using the Preferred Reporting Items for Systematic Reviews and Meta-Analyses guidelines. Relevant studies were identified through searches of databases including PubMed, Embase, and the Cochrane Library, as well as prominent oncology conferences. The search was conducted from the inception of the databases up to January 31, 2024. A total of 10 studies were included in this meta-analysis. Among these studies, six were randomized trials, while four were observational studies. The pooled meta-analysis showed that PD-1 and PD-L1 inhibitors are more effective in improving overall survival and progression-free survival compared to chemotherapy alone. However, when comparing PD-1 and PD-L1 inhibitors, there was no significant difference between the two groups regarding overall survival and progression-free survival. It is important to note that there is no head-to-head trial comparing these two interventions in patients with extensive-stage small-cell lung cancer. Therefore, future prospective trials are needed to define optimal therapeutic approaches in this patient population.

## Introduction and background

Small-cell lung cancer (SCLC) constitutes around 15% of all lung cancer cases and is characterized by its highly invasive and lethal nature [1]. The rapid advancement of this illness leads to almost 70% of individuals being diagnosed with distant metastasis initially, categorizing them as having extensive-stage small-cell lung cancer (ES-SCLC). The outlook for ES-SCLC remains bleak, with a median overall survival (OS) of about 10 months and a post-diagnosis five-year survival rate of approximately 6-7% [2,3]. Despite significant efforts to improve survival outcomes for this patient population, progress has been limited. Etoposide and platinum (EP) combination therapy has been the standard first-line treatment for ES-SCLC for the past three decades [4]. While initial response rates to first-line treatment can reach up to 78% among ES-SCLC patients, these responses are typically short-lived, with the majority experiencing relapse within six months [4].

Given the robust immunogenic characteristics of SCLC cells and the induced release of tumor antigens by chemotherapy, there is considerable anticipation surrounding the combination of immune checkpoint inhibitors (ICIs) and chemotherapy. Nivolumab and pembrolizumab monotherapy, acting as programmed death protein 1 (PD-1) inhibitors, have demonstrated moderate responses in specific SCLC subgroups in initial studies, such as Keynote-028, Checkmate-032, and Keynote-158 trials, prompting their clinical adoption in later-line treatment of metastatic ES-SCLC [5-7].

Furthermore, the programmed death ligand 1 (PD-L1) pathway has shown clinical efficacy when utilized as a first-line treatment in ES-SCLC patients. Noteworthy is the IMpower133 trial, an international phase 3 study, which marked the initial successful demonstration of the efficacy and safety of atezolizumab when combined with carboplatin-etoposide as a primary therapy for ES-SCLC [8]. Similarly, the CASPIAN study, a phase 3 randomized, open-label trial, unveiled consistent and enduring clinical benefits of durvalumab alongside platinum-etoposide as a first-line treatment for ES-SCLC patients, showcasing improvements in OS, progression-free survival (PFS), and objective response compared to a clinically relevant control group [9]. As a result of these pivotal findings, the Food and Drug Administration authorized the use of PD-L1 inhibitors, such as durvalumab and atezolizumab, in conjunction with chemotherapy as the primary treatment regimen for previously untreated patients with ES-SCLC in 2019 and 2020, respectively.

Currently, there exists a significant disparity in the effectiveness and safety profiles of PD-L1 and PD-1 inhibitors. The exact causes for the differences in clinical effectiveness between these two categories of inhibitors are not completely understood. However, one potential explanation could be linked to the interplay between PD-1 and PD-L2, which might impede T-cell activation. PD-1 inhibitors have the capability to disrupt the interaction of PD-1 with both PD-L1 and PD-L2, whereas PD-L1 inhibitors solely block the interaction of PD-1 with PD-L1. Consequently, when administered a PD-L1 inhibitor, tumors could potentially circumvent anti-tumor immune responses via the PD-1/PD-L2 pathway [10,11].

Clinical investigations directly comparing PD-1 and PD-L1 inhibitors are scarce. Therefore, we conducted this systematic review and meta-analysis to ascertain whether the two immunotherapy regimens are comparable in terms of efficacy in patients with ES-SCLC.

## Review

Methodology

The present meta-analysis was conducted using the Preferred Reporting Items for Systematic Reviews and Meta-Analyses (PRISMA) guidelines. Relevant studies were identified through searches of databases, including PubMed, Embase, and the Cochrane Library, as well as prominent oncology conferences such as the American Society of Clinical Oncology, the European Society of Medical Oncology, the American Association for Cancer Research, and the World Conference on Lung Cancer. The search utilized key subject terms such as “immune therapy,” “immunotherapy,” “programmed cell death protein-1,” “programmed cell death-ligand 1,” and “small-cell lung cancer” along with synonyms and medical subject heading terms. The search was conducted from the inception of the databases up to January 31, 2024. Additionally, the bibliographies of included studies were manually screened to identify any additional relevant studies pertaining to the study topic.

Eligibility Criteria

We included all studies (observational or clinical trials) comparing PD-1 or PD-L1 either with each other or with chemotherapy in patients with ES-SCLC. We included studies that enrolled patients aged 18 years or more. Exclusion criteria included studies with no comparison group, those that did not report required outcomes, and animal studies. We also excluded case reports, case series, reviews, and editorials. Two investigators assessed all included studies whether they met the eligibility criteria or not. First, screening was done using titles and abstracts followed by the full-text screening of eligible studies. Studies that met all the inclusion and exclusion criteria were included in the meta-analysis. Screening was performed by two authors independently, and any disagreement between them was resolved through discussion.

Data Collection Procedure

Information from eligible articles was independently gathered by two authors using a standardized data collection sheet (Microsoft Excel, Microsoft Office, 2022). A third reviewer validated the accuracy of the collected data. Extracted data from the included studies encompassed details such as first author, publication year, study design, patient count, drugs used in the study, and outcomes. The outcomes evaluated in this meta-analysis comprised OS and PFS.

Statistical Analysis

We conducted a network meta-analysis (NMA) using RStudio version 4.3.2. For each outcome measure, including OS and PFS, we conducted a random-effects NMA to determine the combined relative effects of interventions. NMA expands upon traditional pairwise meta-analysis by enabling the concurrent comparison of numerous interventions, creating an interconnected network while upholding the internal randomization within individual trials. Random-effects NMA was conducted for each outcome to estimate all conceivable pairwise relative effects and furnish a clinically relevant hierarchy of treatments for advanced-stage SCLC. We depicted summary hazard ratios (HRs) along with their corresponding 95% confidence intervals (CIs) in a heatmap. Moreover, we evaluated the relative hierarchy of treatments for each outcome utilizing the distribution of ranking probabilities and the surface under the cumulative ranking curve (SUCRA). To account for network-specific heterogeneity, we assumed a common heterogeneity parameter for each outcome and estimated predictive intervals to gauge the potential impact of this heterogeneity on relative effects, considering additional uncertainty in future studies. To evaluate statistical inconsistency in the data, indicating disagreement between different sources of evidence, we employed both local and global approaches. The global approach involved calculating regression coefficients of the inconsistency model for each study design and testing the linearity of these coefficients using the Wald test. If the p-value was <0.05 in both global and local tests, we utilized the consistency model; otherwise, inconsistency was employed to compute effect estimates.

Results

**Figure 1:**
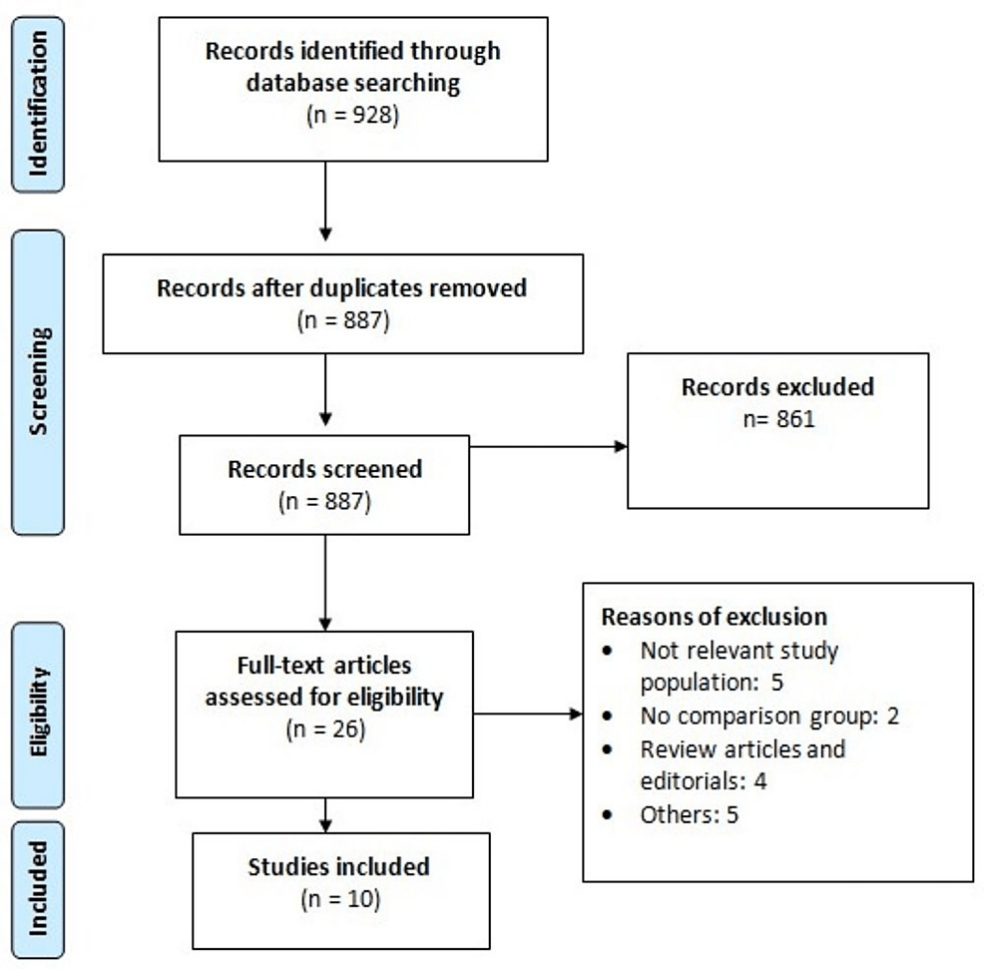
Preferred Reporting Items for Systematic Reviews and Meta-Analyses flowchart of study selection.

Of the 928 records identified through the literature search, screening based on abstract and title was performed on 887 records, followed by full-text screening of 26 articles against the inclusion and exclusion criteria. Ultimately, 10 studies met the eligibility criteria and provided adequate data for inclusion in the meta-analysis. Figure 1 illustrates the PRISMA flowchart detailing the process of study selection. These included studies [9,12-20], published between 2019 and 2024, collectively enrolled a total of 3,231 patients diagnosed with ES-SCLC. Among the included studies, six were randomized trials [9,12,14,15,17,18], while four were [13,16,19,20] observational studies. Table 1 outlines the characteristics of the included studies. In all included studies, PD-1 and PD-L1 were administered with chemotherapy.

**Table 1:** Characteristics of included studies. RCT: randomized control trial; PD-1: programmed cell death protein 1; PD-L1: programmed death ligand 1

Author	Year	Setting	Study design	Region	Groups	Sample size
Cheng et al. [12]	2022	Multicenter	RCT	China	PD-1 group	389
					Placebo	196
Kim et al. [13]	2023	Single center	Observational	Korea	Placebo	48
					Anti-PD-L1 group	41
Leal et al. [14]	2020	Multicenter	RCT	United States	PD-1 group	80
					Placebo	80
Liu et al. [15]	2021	Multicenter	RCT	21 countries	Placebo	196
					PD-L1 group	198
Paz-Ares et al. [9]	2019	Multicenter	RCT	23 countries	Placebo	269
					PD-L1 group	268
Qin et al. [16]	2024	Multicenter	Observational	China	Anti-PD-1 group	68
					Anti-PD-L1 group	86
Rudin et al. [17]	2020	Multicenter	RCT	18 countries	PD-1 group	228
					Placebo	225
Wang et al. [18]	2022	Multicenter	RCT	China	Placebo	232
					Anti-PD-L1 group	230
Wang et al. [19]	2023	Single center	Observational	China	PD-1 group	93
					PD-L1 group	101
Yang et al. [20]	2022	Single center	Observational	China	PD-1 group	146
					PD-L1 group	75

Meta-analysis of outcomes

Overall Survival

**Figure 2:**
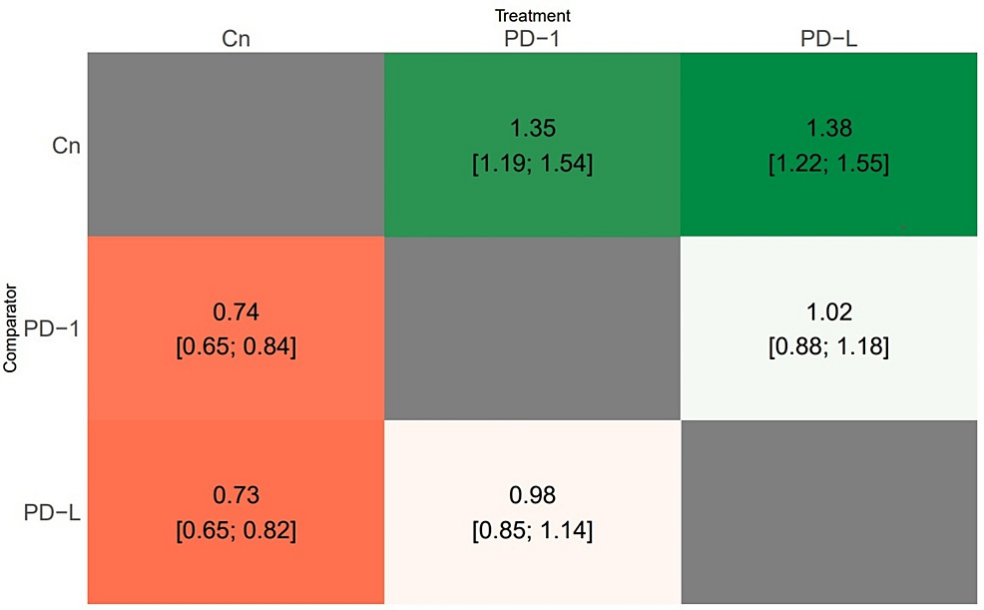
Heat map showing the results of the network meta-analysis comparing the effects of all treatment approaches on overall survival. PD-1: programmed cell death protein 1; PD-L1: programmed cell death ligand 1; Cn: control

A total of 10 studies evaluated this outcome, with three studies comparing PD-1 and PD-L1, three studies comparing PD-1 and a control group, and four studies comparing PD-L1 and a control group. Results from both global and local consistency tests revealed a p-value >0.05, supporting consistency and thus validating the null hypothesis, rendering this network model acceptable. Effect size estimates were obtained for comparisons between each treatment approach regarding OS. Patients administered PD-1 and PD-L1 exhibited significantly improved overall survival compared to those in the control group. However, no significant difference in overall survival was observed between PD-1 and PD-L1 treatments, as shown in Figure 2. Table 2 displays the SUCRA scores for the three treatments. The probability of being deemed the most effective treatment was the highest for PD-L1 (80%), followed by PD-1 (70%).

**Table 2:** SUCRA score. PD-1: programmed cell death protein 1; PD-L1: programmed cell death ligand 1; SUCRA: surface under the cumulative ranking

Treatment groups	Overall survival	Progression-free survival
PD-1	70	87.5
PD-L1	80	62.5
Control	0	0

Progression-Free Survival

**Figure 3:**
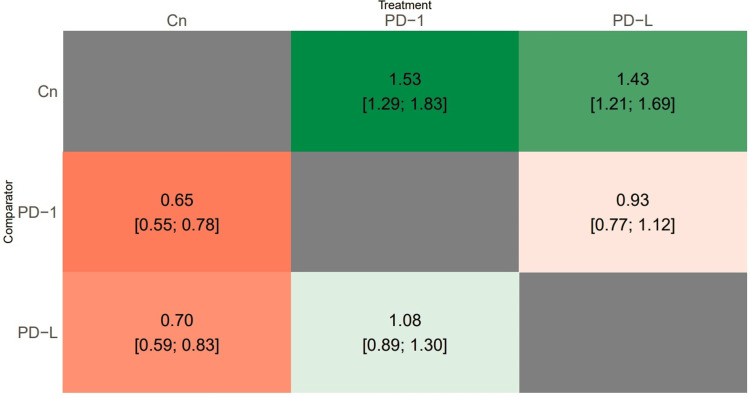
Heat map showing the results of the network meta-analysis comparing the effects of all treatment approaches on progression-free survival. PD-1: programmed cell death protein 1; PD-L1: programmed cell death ligand 1; Cn: control

A total of 10 studies evaluated this outcome, with three studies comparing PD-1 and PD-L1, three studies comparing PD-1 and a control group, and four studies comparing PD-L1 and a control group. Results from both global and local consistency tests indicated a p-value >0.05, supporting consistency and thereby validating the null hypothesis, rendering this network model acceptable. Effect size estimates were derived for comparisons between each treatment approach regarding PFS. Patients administered PD-1 or PD-L1 exhibited significantly improved PFS compared to those in the control group. However, no significant difference in PFS was observed between PD-1 and PD-L1 treatments, as shown in Figure 3. Table 2 displays the SUCRA scores for the three treatments. The probability of being deemed the most effective treatment was the highest for PD-1 (87.5%), followed by PD-L1 (62.5%).

Discussion

Our meta-analysis represents a pioneering effort to assess the comparative effectiveness and safety profiles of PD-1 and PD-L1 inhibitors in patients diagnosed with ES-SCLC. Across 10 studies, including six randomized controlled trials and four observational studies, we conducted a comprehensive evaluation. Our findings from the NMA underscore the significant enhancements in both OS and PFS associated with the combination of PD-1 or PD-L1 inhibitors with chemotherapy compared to chemotherapy alone. Notably, we did not detect statistically significant disparities between PD-1 and PD-L1 inhibitors concerning their impact on OS or PFS.

Prior meta-analyses have also attempted to compare the clinical efficacy and safety profiles of PD-1 inhibitor plus chemotherapy and PD-L1 inhibitor plus chemotherapy regimens [11,21-23]. For instance, Yu et al. found no statistically significant differences in PFS and OS between PD-1 plus chemotherapy and PD-L1 plus chemotherapy combinations [21]. Additionally, Kang et al. conducted an NMA within the Chinese healthcare system, revealing no significant disparities in survival outcomes between the two regimens [22]. While an earlier meta-analysis suggested superior survival outcomes with PD-1 inhibitors compared to PD-L1 inhibitors in solid tumors [23], our study findings align with recent research indicating comparable efficacy of PD-1 and PD-L1 inhibitors in ES-SCLC patients.

However, the mechanisms underlying potential differences between PD-L1 and PD-1 inhibitors in SCLC warrant further investigation, particularly utilizing well-defined animal models. It is clinically imperative to evaluate predictive biomarkers for PD-L1 versus PD-1 inhibitors when administered alongside chemotherapy in SCLC. Despite PD-L1 expression being considered a potential predictive biomarker across various cancer types, its reliability in predicting the efficacy of PD-L1/PD-1 plus chemotherapy in SCLC remains uncertain [15,17]. Challenges in obtaining biopsy samples from SCLC patients, often small and necrotic, underscore the need for innovative approaches to assess biomarkers in this population [15].

While our study suggests similar clinical effects of PD-1 plus chemotherapy and PD-L1 plus chemotherapy, caution is warranted in utilizing PD-1 plus chemotherapy as first-line treatment for ES-SCLC [24]. First, the secondary endpoint status of OS in the EA5161 study raises concerns about its adequacy for regulatory approval. Second, the absence of prospective head-to-head comparisons between PD-L1 plus chemotherapy and PD-1 plus chemotherapy regimens underscores the need for further research in this area. Additionally, the limitations of meta-analysis, including the absence of direct prospective trials comparing PD-L1 and PD-1 inhibitors, should be acknowledged.

Our meta-analysis, despite its contributions, has limitations, including the lack of analysis of baseline data of PD-L1 expression and TMB due to insufficient data. This highlights the necessity for further research to guide the optimal selection of immunotherapy agents in ES-SCLC. While retrospective studies comparing these interventions in ES-SCLC patients were included, prospective trials are essential to validate our findings. Our meta-analysis provides valuable insights into the comparative effectiveness and safety profiles of PD-1 and PD-L1 inhibitors in ES-SCLC patients. While our findings suggest comparable efficacy between the two regimens, further research is warranted to elucidate underlying mechanisms, identify predictive biomarkers, and validate our findings in prospective trials.

## Conclusions

Our meta-analysis comparing PD-1 plus chemotherapy and PD-L1 plus chemotherapy inhibitors in ES-SCLC patients demonstrates similar efficacy in terms of OS and PFS when combined with chemotherapy alone. However, we did not find any significant difference between two groups in terms of OS and PFS. There is no head-to-head trial comparing these two interventions in patients with ES-SCLC. Therefore, we need future prospective trials to define optimal therapeutic approaches in this patient population.
